# Cytochemical flow analysis of intracellular G6PD and aggregate analysis of mosaic G6PD expression

**DOI:** 10.1111/ejh.13013

**Published:** 2018-01-15

**Authors:** Michael Kalnoky, Germana Bancone, Maria Kahn, Cindy S. Chu, Nongnud Chowwiwat, Pornpimon Wilaisrisak, Sampa Pal, Nicole LaRue, Brandon Leader, Francois Nosten, Gonzalo J. Domingo

**Affiliations:** ^1^ Diagnostics Program PATH Seattle WA USA; ^2^ Shoklo Malaria Research Unit Mahidol‐Oxford Tropical Medicine Research Unit Faculty of Tropical Medicine Mahidol University Mae Sot Thailand; ^3^ Centre for Tropical Medicine and Global Health Nuffield Department of Medicine Research building University of Oxford Oxford UK

**Keywords:** G6PD deficiency, hemolytic anemia, lyonization, malaria, *Plasmodium vivax*

## Abstract

**Background:**

Medicines that exert oxidative pressure on red blood cells (RBC) can cause severe hemolysis in patients with glucose‐6‐phosphate dehydrogenase (G6PD) deficiency. Due to X‐chromosome inactivation, females heterozygous for G6PD with 1 allele encoding a G6PD‐deficient protein and the other a normal protein produce 2 RBC populations each expressing exclusively 1 allele. The G6PD mosaic is not captured with routine G6PD tests.

**Methods:**

An open‐source software tool for G6PD cytofluorometric data interpretation is described. The tool interprets data in terms of % bright RBC, or cells with normal G6PD activity in specimens collected from 2 geographically and ethnically distinct populations, an African American cohort (USA) and a Karen and Burman ethnic cohort (Thailand) comprising 242 specimens including 89 heterozygous females.

**Results:**

The tool allowed comparison of data across 2 laboratories and both populations. Hemizygous normal or deficient males and homozygous normal or deficient females cluster at narrow % bright cells with mean values of 96%, or 6% (males) and 97%, or 2% (females), respectively. Heterozygous females show a distribution of 10‐85% bright cells and a mean of 50%. The distributions are associated with the severity of the G6PD mutation.

**Conclusions:**

Consistent cytofluorometric G6PD analysis facilitates interlaboratory comparison of cellular G6PD profiles and contributes to understanding primaquine‐associated hemolytic risk.

## 1 INTRODUCTION

Glucose‐6‐phosphate dehydrogenase deficiency affects more than 400 million people or 8% of the general population of malaria‐endemic nations.^1^, ^2^, ^3^ The enzyme G6PD is required in red blood cells to protect the red blood cells against oxidative challenges.^4^ Specifically, 8‐aminoquinolines, currently the only class of drugs that can totally cure a patient of *Plasmodium vivax*, submit red blood cells to oxidative stress in a dose‐dependent way. Patients with deficiency in G6PD activity are at risk of suffering severe hemolysis when exposed to the high doses of primaquine required for radical cure. Consequently, the WHO recommends testing for G6PD deficiency when possible prior to administering curative doses of primaquine.^5^

Glucose‐6‐phosphate dehydrogenase (G6PD) deficiency is an inherited, X‐linked trait.^6^, ^7^ Hemizygous males and homozygous females are either severely G6PD deficient or normal depending on whether they have wild‐type alleles or alleles that encode for G6PD enzyme with compromised enzyme stability and activity. Heterozygous females with 1 normal allele and 1 mutated allele present with a broader range of G6PD activity. The random inactivation of one or the other X chromosomes during embryonic development (lyonization) results in females having populations of red blood cells expressing G6PD deficiency in fixed proportions ranging typically from 20 to 80%.^7^, ^8^

Safe case management of *P. vivax*, with 8‐aminoquinolines, requires knowledge of the G6PD status of the patient to prevent severe hemolytic anemia. In cases where G6PD activity is too low, 8‐aminoquinolines should not be administered. Several quantitative and qualitative assays are available for the diagnosis of G6PD deficiency through measurement of residual G6PD activity in whole blood.^9^, ^10^ Qualitative tests cannot, however, stratify women with intermediate activity above 30‐40% normal G6PD activity, and quantitative tests do not provide information regarding relative ratios of allele representation in a heterozygous female red blood cell population.

Cytochemical staining of red blood cells followed by observation with either microscopy or flow cytometry represents the only way to observe the mosaic red blood cell population in heterozygous females.^11^, ^12^, ^13^, ^14^, ^15^, ^16^, ^17^ Cytofluorometric assays represent an opportunity to determine the relative G6PD activity at the level of the individual red blood cell. Recent development of complementary methodologies makes these assays more robust for wider use.^18^, ^19^ Thus, it is possible to identify females with distinct populations of RBCs expressing either a G6PD‐deficient allele or a normal one, thereby measuring intermediate G6PD activity in heterozygous females, but so far there is no method to standardize this process. Arbitrary threshold setting in cytofluorometry assay can be subjective, thereby influencing how percentage of G6PD normal cells is designated. Here, a new software tool which provides automated analysis of percent G6PD normal cells and removes operator and instrument variation is presented. The software tool is a Web‐based tool that does not require any programming skills. The tool can be used to standardize interpretation of cytofluorometric data. Additionally, 2 datasets, 1 from Thailand and 1 from the USA, are used to demonstrate the utility of the tool for determination of heterozygosity for G6PD in females. Association between mosaic profiles, G6PD activity, and hemoglobinopathies has been published separately.^12^, ^20^, ^21^, ^22^

## 2 MATERIALS AND METHODS

### 2.1 Human subjects research and specimen handling

US donor blood specimens were obtained by Bioreclamation, Inc. (Westbury, NY, USA) and were collected between January 2012 and January 2016 from volunteers who were at least 18 years of age and who signed consent under institutional review board protocol by the Schulman IRB (Cincinnati, OH, USA), 2010‐017 IRB. All donors were of African American origin, presenting at a recruitment center in New York, USA. Specimens were transported in ethylenediaminetetraacetic acid (EDTA) anticoagulant venipuncture vacuum tubes on cold packs and were stored at 4°C. Specimen processing took place between 2 and 4 days after blood collection. Tests for a given comparison typically were conducted on the same day for each blood sample. No personal identification data were collected, and all G6PD assays were performed independently and blinded to G6PD status.

Specimens from Thailand were collected by Shoklo Malaria Research Unit (SMRU, Mae Sot, Tak Province) from volunteers between February and April 2014. Donors were from migrant populations residing along the Thailand‐Myanmar border composed of Burman and Karen ethnic groups as described previously.^20^ Healthy volunteers over 18 years were recruited at the field clinics. After written informed consent was obtained, blood was collected and the samples were refrigerated for a maximum of 6 hours and shipped in cool boxes to the central hematology laboratory at SMRU where research procedures were conducted. Ethical approvals for this study were obtained from the Mahidol University Faculty of Tropical Medicine Ethics Committee (FTMEC), Oxford Tropical Research Ethics Committee (OxTREC), and the PATH Research Ethics Committee (REC). The protocol was also reviewed by the Community Advisory Board at SMRU, which is composed of representatives from the communities served by SMRU. Volunteers who met the inclusion criteria underwent a detailed informed consent process and provided written consent before enrolling in the study.

In total, specimens from 242 volunteer donors were included in the analysis reported here: 97 from the USA, of which 49 were females and 48 were males, and 145 from Thailand, of which 95 were females and 50 were males.

### 2.2 G6PD activity

All specimens were characterized for G6PD activity in duplicate with the quantitative G6PD kit from Trinity Biotech (Cat. No. 345‐B; Trinity Biotech PLC, Bray, Co. Wicklow, Ireland) according to manufacturer's instructions, as the reference assay for all testing, as described previously.^12^, ^20^ Normal, intermediate, and deficient Trinity controls (Cat. Nos. G6888, G5029, G5888, respectively) were run using the same method on each day of testing. Enzyme activity was determined using a temperature‐regulated spectrophotometer (UV‐1800 Shimadzu, Shimadzu Scientific Instruments, Columbia, MD, USA) set at 30°C, by measuring the change in rate in absorbance at 340 nm over 5 minutes. G6PD activity values were calculated in units per gram of hemoglobin (U/g Hb). Hemoglobin concentration was determined using a HemoCue hemoglobin system (HemoCue™ Hb 201 +  Analyzer, No. 121721, Cat. No. 22‐601‐007; Fisher Scientific, Inc., Waltham, MA, USA) or by automated hematology analyzer (CeltacF MEK‐8222K, Nihon Kohden, Japan).

### 2.3 G6PD genotyping

The G6PD sequence for all specimens used in this report was confirmed by DNA sequencing as described previously.^12^ Single nucleotide polymorphisms (SNPs) and insertion‐deletions (INDELS) were determined using the Genome Analysis Toolkit (GATK) (Broad Institute, Cambridge, MA, USA). The DNA libraries from genomic DNA (gDNA) of proband and controls were constructed according to Illumina paired‐end libraries construction protocol. A custom design array, which contains all the exon sequences and their flanking sequences of the G6PD, was used in this study; after hybridization and washing, sequencing was then performed with the HiSeq2000 (Illumina, San Diego, CA, USA). Image analysis and base calling were performed using the Illumina Pipeline (version 1.3.4) to generate primary data.

### 2.4 Cytofluorometry

Whole blood specimens were characterized for intracellular G6PD activity by flow cytometry as described previously.^15^ Ten microliters of 50% hematocrit red blood cell suspension was diluted into 90 μL of 0.9% NaCl and was combined with 100 μL of sodium nitrite (0.125 mol/L, MilliporeSigma, St.Louis, MO, USA) and incubated at room temperature for 20 minutes. Samples were washed 3 times with phosphate‐buffered saline (PBS) at 1000 *g* for 3 minutes and resuspended in 100 μL of PBS. The red blood cells were then combined with 18 μL of glucose (0.28 mol/L) in phosphate‐buffered saline and 6 μL of Nile blue sulfate (0.01% Sigma) and incubated at 37°C for 90 minutes using open lid Eppendorf tubes. After the incubation, 2.5 μL of 0.4 mol/L potassium cyanide (Sigma) was added and incubated for 5 minutes. Five microliters of each sample was added to 100 μL of 3% hydrogen peroxide in PBS, agitated vigorously by hand, and washed 2 times in PBS. Specimens were analyzed using an Accuri™ C6‐UV flow cytometer (BD Biosciences, San Jose, CA, USA) on a total of 30 000 RBCs per replicate in the FL1 channel 533 ± 30 nm.

### 2.5 Software tool development

The mathematical algorithm and graphical user interface (GUI) as well as the validation of the analysis tool were developed using the statistical software “R” (http://www.r-project.org/) (R Foundation for statistical computing, Vienna, Austria). Custom scripts were written to generate the algorithm and GUI as well as implementation of the R flowCore package in R for the importation of flow cytometry data as well as R shiny package for building the graphical user interface. The flowCore package was developed at Bioconductor (WA, USA).^23^ Shiny is a Web application framework for the R programming environment.^24^ The G6PD flow data analysis tool named mosaic G6PD flow is freely accessible to all users on the Shiny server.^25^

### 2.6 Statistical methods

All statistical analyses were conducted in the open‐source statistical computing language “R” (http://www.r-project.org/). The sensitivities, and specificities, of the cytofluorometric assay were calculated using DNA sequencing as a reference standard.

## 3 RESULTS

### 3.1 Genotypic characterization of blood specimens

The G6PD alleles for all specimens were sequenced, with a summary provided in Table 1. In the US African American sample, 31 of a total of 97 donors were confirmed heterozygous females. In the Thai sample set, 58 of a total of 145 specimens were confirmed heterozygous females. All heterozygous females except three had a Mahidol G6PD‐deficient allele, and the 3 remaining had Mediterranean, Kaiping, and a newly found allele called “Shoklo.” One deficient homozygous woman was carrier of 2 different mutations (Mahidol and Orissa), and the remaining homozygous were Mahidol. Likewise, all hemizygous G6PD‐deficient males were Mahidol except one which was Viangchan.^21^

**Table 1:** Summary of study genotypes for the US and Thailand cohorts as determined by DNA sequencing

Cluster	Genotype	Mutation	Amino acid substitution	N
US COHORT				
Males				
(+)	Hemizygous	Normal	‐	23
(+)	Hemizygous	A+	N126D	6
(−)	Hemizygous	A−^(202)^	N126D and V68M	19
Females				
(+_1_/+_1_)	Homozygous	Normal	‐	18
(+_1_/+_2_)	Heterozygous	Normal/A+	‐/N126D	14
(+/−)	Heterozygous	Normal/A−^(202)^	‐/N126D and V68M	17
Thailand cohort				
Males				
(+)	Hemizygous	Normal	‐	26
(−)	Hemizygous	Viangchan	G291A	1
(−)	Hemizygous	Mahidol	G163S	23
Females				
(+_1_/+_1_)	Homozygous	Normal	‐	20
(+/−)	Heterozygous	Mahidol	G163S	55
(−_1_/−_2_)	Heterozygous	Orissa Mahidol	C44G and G163S	1
(+/−)	Heterozygous	Kaiping	G1388A	1
(+/−)	Heterozygous	Medit	C188T	1
(−_1_/−_1_)	Homozygous	Mahidol	G163S	17

### 3.2 Characterization of blood specimens by G6PD enzyme activity assay

All specimens were characterized for G6PD activity with the quantitative G6PD kit from Trinity Biotech. The descriptive analysis for the G6PD activity for the different genotypes is provided in Table 2. The association between G6PD activity and different hematological characteristics is described elsewhere.^21^

**Table 2:** Descriptive statistics for glucose‐6‐phosphate dehydrogenase (G6PD) activity arranged by G6PD genotype described in terms of minimum (Min.), median, mean, standard deviation (SD), and maximum (Max.)

	Min.	Median	Mean	SD	Max.
Males					
Hemizygous^a^ WT (+)	6.7	8.7	9.3	2.0	14.0
Hemizygous^a^ A+ (+)	6.6	8.3	8.3	1.7	11.4
Hemizygous^a^ (−)	0.7	1.3	1.3	0.3	1.9
Hemizygous^b^ WT(+)	6.5	7.5	7.7	1.1	10.8
Hemizygous^b^ (+)	0.6	1.0	1.0	0.3	1.6
Females					
Homozygous^a^ WT (+_1_/+_1_)	6.2	9.5	9.4	1.6	12.4
Heterozygous^a^ A+ (+_1_/+_2_)	6.8	8.5	8.6	1.4	11.3
Heterozygous^a^ (+/−)	3.0	5.8	5.6	1.5	7.6
Homozygous^b^ WT (+_1_/+_1_)	5.2	7.5	7.9	1.8	12.7
Heterozygous^b^ (+/−)	0.7	4.1	4.1	1.4	7.2
Homozygous^b^ (−/−)	0.7	1.3	1.3	0.4	2.4

The statistics are shown only for the genotypes for which there were more than 1 representative specimen in the sample set.

a For the US sample set.

b For the Thailand sample set.

### 3.3 G6PD normal to G6PD‐deficient red blood cell ratio calculation

The cytofluorometric method allows observation of mosaic red blood cell populations in specimens from females by looking at the activity of G6PD in individual erythrocytes. An algorithm was developed to standardize interpretation of cytofluorometric data.^25^

**Figure 1:**
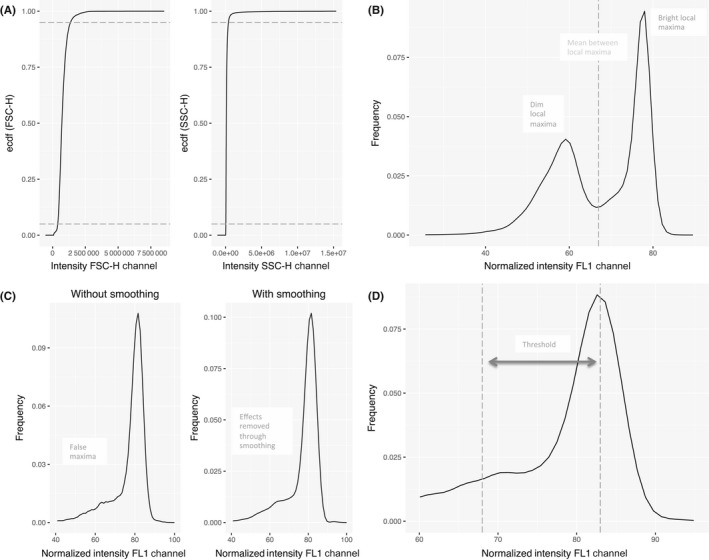
Process for standardized interpretation of cytofluorometric intrared blood cell glucose‐6‐phosphate dehydrogenase (G6PD) data. Data processing is shown for a heterozygous specimen (panels A and B) and a normal hemizygous male (panels C and D). A, Removal of lower and upper 5% of forward scattered count (FSC) and side scatter counts (SSC). The empirical cumulative distribution function for the FCS and SSC, respectively, for a clinical specimen is shown. B, After normalization of the data and generation of kernel density estimations, an algorithm is applied to identify peak maxima associated with dim (G6PD deficient) and bright (G6PD normal) red blood cells. C, Data are smoothed to remove small artifacts. The normalized intensity versus frequency for FL1 channel is shown pre‐ and postsmoothing. D, A hard cutoff threshold is applied to the standardized and smoothed data to allow for only maximal peak

The algorithm for doing this is summarized in the following steps (Figure 1):

Step 1: Correct selection of amplification mode (log or linear) and the correct channel numbers are assigned to FL1, forward scattered count (FSC), and side scatter counts (SSC).
Step 2: QC—After import of.fcs file, the lower and upper 5% of FSC and SSC values are truncated. This helps remove unhealthy cells from the sample population (Figure 1A).
Step 3: Standardization—A scaling algorithm is applied to data to account for variation from different cytometers due to differences in set gain and amplification. All new data are adjusted for gain and amplification effects to fit a standard of fluorescence ranges from 0 to 1000.
Step 4: Kernal density estimation—For each set of remaining FITC values, an estimation of the distribution of intensity is performed using a kernal density estimation method with a Gaussian kernal. Resulting histogram data are converted into a probability distribution function for analysis of features. An algorithm searches for local maxima corresponding to dim and bright peaks (Figure 1B).
Step 5: Data smoothing—A smoothing function is applied to the distribution of intensity obtained from kernal density estimation. The smoothing function removes small artifacts in the data and leaves only the major features of the distribution (Figure 1C).
Step 6: Thresholding—A hard cutoff threshold is applied to the standardized and smoothed data. Peaks that are too close to one another are treated so that smaller secondary peaks within a 15% margin would not be counted as a separate peak (either dim or bright) based on local maxima (Figure 1D).
Step 7: Data interpretation—The locations of local maxima are determined using a threshold technique and evaluated to determine the proportions of dim and bright cells. The local maxima are automatically calculated when a file is uploaded. The results are displayed graphically in the visualizations tab.

### 3.4 The Web browser accessible software tool for G6PD cytofluorometric data normalization and interpretation

**Figure 2:**
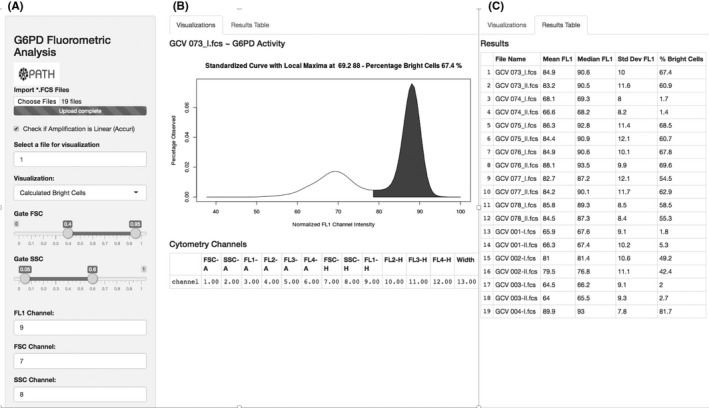
Graphical user interface for software tool to normalize glucose‐6‐phosphate dehydrogenase (G6PD) cytofluorometric data and standardize its interpretation. A, Sidebar from where files can be imported and channels selected. B, Visualization of the bright and dim cell population for each individual specimen dataset. C, Results panel showing values for mean, median, and standard deviation of the FL1 channel as well as the % bright cells for each specimen. This table can be downloaded as a csv file using the download button in the sidebar A

A software tool publically available on the Web browser was developed to allow users running the same cytofluorometric assay analyze data in the same way.^15^ The resulting graphical user interface (GUI) contains a sidebar and 2 tabs as shown in Figure 2. Panel A is the sidebar from where file can be imported and channels can be selected. Panel B helps the visualization of the bright and dim cell population. Panel C tabulates values for mean, median, and standard deviation of the FL1 channel as well as the % bright cells for each specimen. This table can be downloaded using the download button in the sidebar. Data uploaded into the tool are not stored beyond the user's interaction with the tool. When the Webpage that hosts the software tool is refreshed, all previously uploaded data that are used for analysis are deleted from the memory.

### 3.5 Relative portions of normal to deficient cells across 2 diverse populations

**Figure 3:**
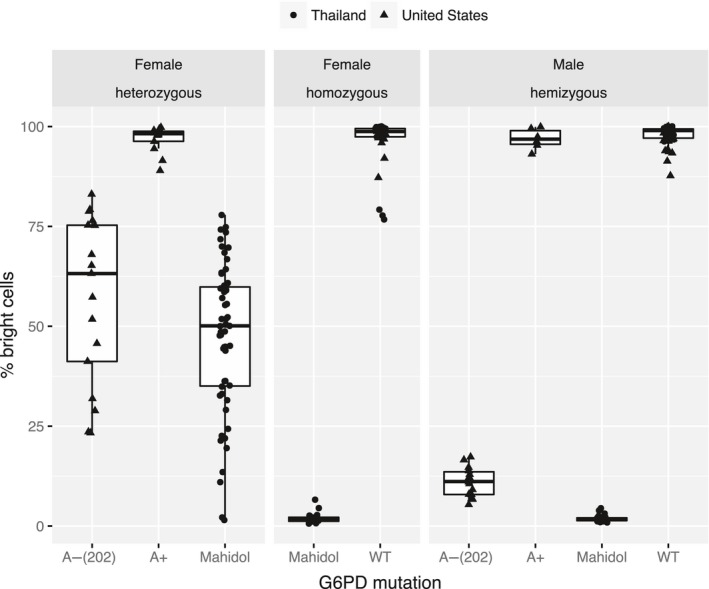
Percent bright cells (glucose‐6‐phosphate dehydrogenase [G6PD] normal) per gender and G6PD mutations. Box plots for the distributions of % percent bright cells observed per specimen per G6PD genotype are shown highlighting minimum and maximum (whiskers), 1st quartile and 3rd quartiles (boxes), and means. The distributions are shown only for the genotypes for which there were more than 1 representative specimen in the sample set. The statistics are described in Table 3

The normalization process for G6PD cytofluorometric data as described above was applied to data collected from donors on the Thailand/Myanmar border and in New York, USA. All specimens were collected from healthy adults and had accompanying DNA sequence and reference quantitative G6PD activity associated with them. There was 100% genotype to phenotype (by the quantitative G6PD assay) concordance (data not shown). The distributions of % bright cells resulting from the standardized flow data analysis for the different genotypes are shown in Figure 3 and described in Table 3.

**Table 3:** Distribution of percent bright cells observed per specimen per glucose‐6‐phosphate dehydrogenase (G6PD) genotype described in terms of minimum (Min.), 1st quartile (1st Qu.), median, mean, 3rd quartile (3rd Qu.), and maximum

	Min.	1st Qu.	Median	Mean	3rd Qu.	Max.
Males						
Hemizygous WT (+)	87.7	97.1	99	97.92	99.4	100
Hemizygous A+ (+)	93.1	95.55	96.85	96.92	98.98	99.9
Hemizygous A− [202] (−)	5.4	7.9	11.15	10.92	13.58	17.3
Hemizygous Mahidol (−)	0.9	1.35	1.8	1.913	2.05	4.5
Females						
Heterozygous A− (202) (+/−)	23.3	41.2	63.2	56.95	75.3	83.1
Heterozygous Mahidol (+/−)	1.5	35.05	50.1	47.32	59.85	77.9
Heterozygous A+ (+_1_/+_2_)	89	96.3	98.2	96.87	98.8	99.8
Homozygous WT (+_1_/+_1_)	76.7	97.43	98.75	96.65	99.5	100
Homozygous Mahidol (−_1_/−_1_)	0.6	1.2	1.6	2.035	2.2	6.6

The statistics are shown only for the genotypes for which there were more than 1 representative specimen in the sample set. This distribution is represented in Figure 3.

### 3.6 Interpretation of normalized cytofluorometric data to determine allele composition

The primary metric in determining allele composition from the cytochemical assay is determining the proportion of cells which correspond to cell populations with normal G6PD activity. For purposes of analysis of the data for determining allele composition, the different genotypes were categorized into 5 clusters: (1) male hemizygous normal (+), (2) male hemizygous deficient (−), (3) female heterozygous normal/deficient (+/−), (4) female normal, including homozygous normal (+_1_/+_1_) and heterozygous normal (+_1_/+_2_), and (5) female deficient, including homozygous deficient (−_1_/−_1_) and heterozygous deficient (−_1_/−_2_).

**Figure 4:**
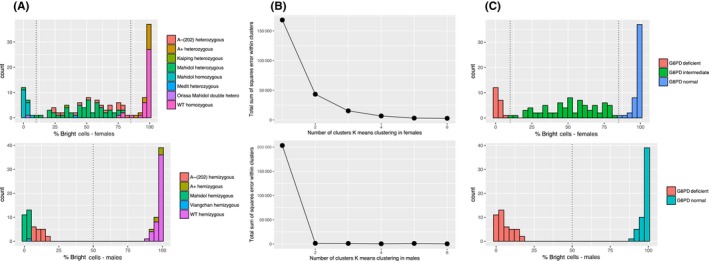
Histogram of bright cells for combined US and Thai samples from Figure 3 used to visualize natural clustering of samples by glucose‐6‐phosphate dehydrogenase (G6PD) phenotype when percent bright cells metric is used and generated from normalized cytofluorometric data. A, Histograms for female and male data (B) elbow method displaying optimal clusters in a K means clustering algorithm. C, Categorization of G6PD phenotypes for males and females using K means algorithm and optimal number of clusters [Colour figure can be viewed at http://wileyonlinelibrary.com]

Figure 4A displays the distribution of percent bright cells for female and male samples in the US and Thai datasets combined. In the case of the female samples, the genotypes leading to G6PD deficient, intermediate, and normal phenotypes cluster nicely from left to right using percent bright cells as the metric to measure G6PD activity. Similarly, the male samples cluster nicely by deficient and normal phenotypes. To confirm this more rigorously, a K means clustering algorithm using from 1 to 6 clusters was applied and the variance was observed as a function of the number of clusters (Figure 4B). Applying the elbow method for optimal clusters, the optimal number of clusters for females and males is 3 and 2, respectively. Figure 4C shows that when the female and male data are categorized into 3 and 2 clusters, the data roughly cluster as expected by G6PD phenotype which indicates that the metric of percent bright cells may be a suitable method for determining G6PD intermediate activity phenotypes. Another approach would be to generate thresholds in percent bright cells visually from the data and apply these thresholds as a categorization method. In this scenario, a female sample with percent bright cells between 10 and 85 percent may be characterized as a sample with intermediate G6PD activity. Preliminary visual thresholds for determining G6PD activity are tabulated in Table 4.

**Table 4:** Ranges of percent bright cells for the different glucose‐6‐phosphate dehydrogenase (G6PD) genotypes established through visual analysis of the standardize flow from combined Thai/US sample set (total 242 samples)

Zygosity		% Bright cells
Males		
Hemizygous normal	(+)	50%‐100%
Hemizygous deficient	(−)	0%‐50%
Females		
Homozygous normal	(+_1_/+_1_)	85%‐100%
Heterozygous normal	(+_1_/+_2_)	
Heterozygous normal/deficient	(+/−)	10%‐85%
Heterozygous deficient	(−_1_/−_2_)	0%‐10%
Homozygous deficient	(−_1_/−_1_)	

## 4 DISCUSSION

DNA sequencing provides the only way to determine definitively the G6PD allele composition both in males and in females. Sequencing can only reliably predict the phenotype in males and for those females with either 2 normal G6PD alleles or 2 deficient G6PD alleles. Sequencing cannot provide any phenotypic information in the case of females with 1 G6PD normal and 1 G6PD‐deficient allele as a result of lyonization.^4^, ^7^, ^8^ Labeling of individual red blood cells for G6PD activity and direct observation by microscopy or flow cytometry provides the only means to determine accurately the relative representation of the expression of the 2 alleles (normal and deficient) in the red blood cells.^14^, ^15^, ^16^ These methodologies are not currently used as diagnostic tests, given their complexity and involvement of toxic chemicals, but as they become more robust, as research tools, they can provide useful information toward the understanding of G6PD activity‐related hemolytic risk. One current limitation for these methodologies is that interpretation of the data in terms of % bright cells as a proxy for percent of red blood cells with normal levels of G6PD activity relies on an arbitrarily set threshold of signal above which cells are defined as normal, making interlaboratory and report comparisons of data challenging.^12^, ^13^ This article presents a publically available software tool for automated and standardized G6PD flow data analysis.

Two hundred and forty‐two blood samples, collected from 2 geographically distinct donor populations and processed in 2 distinct laboratories, were analyzed for G6PD status by DNA sequencing, G6PD enzyme activity, and intracellular RBC G6PD activity by flow cytometry. The comparison of enzyme activity to genotype and intracellular RBC G6PD activity profiles has been described elsewhere.^12^, ^20^, ^21^, ^22^ In this methods article, using a quantitative method, percent bright cells, generated from the same cytofluorometric assay, are compared to results from DNA sequencing. This sample set includes 86 heterozygous females and the deficiency traits, Vianchang, Kaiping, Mediterranean, as A‐ and Mahidol. An algorithm was developed to standardize and normalize the cytofluorometric data across both laboratories and generate a normalized value for % bright cells for each specimen. This analysis has been packaged into publically available software that automates data analysis for the G6PD cytofluorometric assay and allows for standardization of data across different flow cytometers.

Analysis of the cytofluorometric data confirms the diverse ranges of bright (G6PD normal) to dim (G6PD deficient) red blood cells in heterozygous females, ranging from 1 to 84% bright cells, however, with a mean of approximately 50% bright cells. Hemizygous normal or deficient males or females with both alleles either normal for G6PD or deficient for G6PD clustered at much narrower % bright cells ranges with mean % bright cells of 96%, and 6% for males and 97%, and 2% females, respectively.

Visual exploratory analysis and K means clustering analysis seem to indicate the metric of percent bright cells derived from normalized flow cytometry data that may serve as a good metric to categorize samples by G6PD phenotype for males and females. However, using this methodology there are times in which females with normal G6PD alleles may be categorized as intermediate G6PD activity samples. There may be 3 reasons that contribute to females who are normal by G6PD allele but may be categorized as heterozygous by the flow data: (i) genuine biology, in that these women may have a relatively high percent of G6PD‐deficient red blood cells; (ii) compromising of the specimen integrity, it has been observed that the flow assay is highly sensitive to how the specimen is handled postcollection; and (iii) limitations of the tool in analysis of the flow data. Formulations have been developed to improve specimen handling for cytofluorometric G6PD assays,^18^, ^19^ and further data across laboratories and G6PD genotypes will have to be analyzed to further validate the thresholds described here.

It should be noted that the % bright cell output is not strictly an accurate numeration of the 2 allele representation in the red blood cell population in heterozygous females. This is because the red blood cell distributions from the deficient subjects also have red blood cells with normal G6PD levels (contributing to the bright cell portion), and likewise, the G6PD normal subjects have old red blood cells with low intracellular G6PD in the dim cell portion. The Mahidol allele is considered to confer a more severe G6PD deficiency phenotype than the A‐ allele. Correspondingly, the Mahidol male hemizygous‐deficient G6PD cell profiles seem to be more polarized toward low numbers of G6PD bright cells as compared to the A‐ population, and the same is also observed for females heterozygous for the Mahidol allele versus A‐ allele although in both cases the *t* tests were not strongly significant (0.2 and 0.052, respectively).

In conclusion, a publically available Web browser‐based tool with no requirements for programming skills has been developed to standardize analysis of cytofluorometric G6PD data. This tool allows interlaboratory data comparison and broader cross‐geographical and genotypic analysis of G6PD deficiency, especially in females.^21^ An important next step is to attempt to associate this phenotypic data to risk of hemolysis on exposure to an oxidative stress such as primaquine and its metabolites.

## ETHICS APPROVAL AND CONSENT TO PARTICIPATE

For the US study participants, blood specimens were obtained by Bioreclamation, Inc. (Westbury, NY, USA) and were collected between January 2012 and January 2016 from volunteers who were at least 18 years of age and who signed consent under institutional review board protocol by the Schulman IRB (Cincinnati, OH, USA), 2010‐017 IRB.

For the Thailand study, ethics approvals were obtained from the Mahidol University Faculty of Tropical Medicine Ethics Committee (FTMEC), Oxford Tropical Research Ethics Committee (OxTREC), and the PATH Research Ethics Committee (REC). The protocol was also reviewed by the Community Advisory Board at SMRU, which is composed of representatives from the communities served by SMRU. Volunteers who met the inclusion criteria underwent a detailed informed consent process and provided written consent before enrolling in the study.

## CONSENT FOR PUBLICATION

Not applicable.

## AVAILABILITY OF DATA AND MATERIAL

All DNA sequence data have been made available on the NCBI Genbank. The software tool described in this article is publically available at https://mkalnoky.shinyapps.io/MosaicG6PDflow/.

## COMPETING INTERESTS

The authors declare that they have no competing interests.

## AUTHORS' CONTRIBUTIONS

MKalnoky performed the cytofluorometric data analysis, development of algorithms and software. MKhan and NLR performed the US specimen handling and G6PD assay. BL performed all US DNA genotyping analysis. GB, NC, and PW performed all Thailand specimen handling and G6PD characterization. SP, CC, FN, and GJD contributed to the study design, experimental design, and the coordination and write up of the manuscript. All authors contributed to writing of the manuscript. All authors read and approved the final manuscript. All authors declare no competing financial interests.
